# Supplementary material for: Towards unified AI-driven fracture mechanics: the extended deep energy method (XDEM)
Source: Nat Commun. 2026 Aug 15;17:8492. doi: 10.1038/s41467-026-76748-1 (PMC13484471; doi:10.1038/s41467-026-76748-1)
Supplement: Supplementary file 3 [file 41467_2026_76748_MOESM3_ESM.zip › pic/Results/Crack_propagation/Straight/2D_dis_force/results/FEM/μû¡Φúéτ¢╕σ£║μ│òUEL+UMATσ«₧τÄ░.pdf]

改子程序流程：

1. 修改 N_ELEM 相场单元个数，UMAT 和 UEL 都要改

调整 park 相场折减常数 PROPS(four)

STATEV 索引	写入源头	物理含义	在哪个 UEL 分支写入
1	位移单元	积分点位移 u_x	JTYPE = 3/4
2	位移单元	积分点位移 u_y	〃
3	位移单元	ε_{xx} (平面应变)	〃
4	位移单元	ε_{yy}	〃
5	位移单元	γ_{xy} = 2ε_{xy}	〃
6	位移单元	退化后 σ_{xx} · g(φ)	〃
7	位移单元	退化后 σ_{yy} · g(φ)	〃
8	位移单元	退化后 σ_{xy} · g(φ)	〃
9	位移单元	σ_{xx} (纯弹性)	〃
10	位移单元	σ_{yy} (纯弹性)	〃
11	位移单元	σ_{xy} (纯弹性)	〃
12	位移单元	退化后弹性能 g(φ)·ψ_{el}	〃
13	位移单元	纯弹性能 ψ_{el}	〃
14	位移单元 ← 相场单元	φ（当前迭代）	读自相场，写在位移
15	相场单元	φ（上一时间步收敛）	JTYPE = 1/2
16	相场单元	最大历史弹性能 H	〃

编了三套单元

1. 求相场
2. 求位移
3. UMAT 用

所有单元共用节点，边界条件加到节点上。

改 inp 流程：

1. 节点后加四种单元类型

```
*Part, name=plane_strain
*Node
      1,          0.,          0.
      2,          1.,          0.
      3,          0.,          1.
      4,          1.,          1.
********************** TRIANGULAR ***************************
*User element, nodes=3, type=U1, properties=3, coordinates=2, VARIABLES=6
3
**************************************************************
*User element, nodes=3, type=U3, properties=4, coordinates=2, VARIABLES=42
1,2
**************************************************************
********************** RECTENGULAR **************************
*User element, nodes=4, type=U2, properties=3, coordinates=2, VARIABLES=8
3
**************************************************************
*User element, nodes=4, type=U4, properties=4, coordinates=2, VARIABLES=56
1,2
```

其中 1，2 或 3 是自由度

2. 分别给 U1、U2、U3、U4 指定单元

```
********************** TRIANGULAR ***************************
*User element, nodes=3, type=U1, properties=3, coordinates=2, VARIABLES=6
3
**************************************************************
*User element, nodes=3, type=U3, properties=4, coordinates=2, VARIABLES=42
1,2
**************************************************************
********************** RECTENGULAR **************************
*User element, nodes=4, type=U2, properties=3, coordinates=2, VARIABLES=8
3
**************************************************************
*User element, nodes=4, type=U4, properties=4, coordinates=2, VARIABLES=56
1,2

*Element, type=U2
1, 1, 2, 4, 3

*Element, type=U4
2, 1, 2, 4, 3
```

3. 指定材料属性

```
*Element, type=U2
1, 1, 2, 4, 3

*Element, type=U4
2, 1, 2, 4, 3

********************* ASSIGNING MATERIAL PROP ****************
*Elset, elset=SampleSET, generate
   1,  1,    1

*Uel property, elset=SampleSET
0.01, 0.0027, 1
**lc, gc, h

*Elset, elset=SampleSET_SS, generate
2, 2, 1
**************************************************************
*Uel property, elset=SampleSET_SS
210, 0.3, 1, 1e-07
```

从 1 到 1，间隔 1 的所有单元赋予 UEL 的相场单元 l0、Gc 和厚度 h 属性

从 2 到 2，间隔 1 的所有单元赋予 UEL 的弹性单元 E、v、k 属性，k 是折减中的场系数

4. 给 UMAT 赋予单元类型和材料属性，并结束 part

```
******************************************************************
*Element, type=CPE4
3, 1, 2, 4, 3

******************************************************************
*Elset, elset=umatelem, generate
3, 3, 1
```

```
******************************************************************
*Solid Section, elset=umatelem, material=UMATELEM
1.0
```

厚度 1.0

```
*Solid Section, elset=umatelem, material=UMATELEM
1.0
*End Part
```

5. 装配， 指定 instance

```
** ASSEMBLY
**
*Assembly, name=Assemblya
**
*Instance, name=plane_strain-1, part=plane_strain
*End Instance
```

6. 指定加载或边界条件的节点为集合，方便后面加载或施加边界条件（可选）

或施加耦合方程便于加载也在这个位置。 （应力集中的问题似乎可以通过耦合并加载的方式解决）

然后结束装配。Amplitude 也在结束装配后创建。

7. 给 UMAT 单元赋予属性，要很小的刚度

```
*End Assembly
**
** MATERIALS
**
*Material, name=UMATELEM
*Depvar
    16,
*User Material, constants=2
 1e-11, 0.3
**
```

8. 边界条件和分析步

分析步要输出 SDV、RF 和 U

```
*User Material, constants=2
 1e-11, 0.3
**
** BOUNDARY CONDITIONS
**
** Name: bottom_fix Type: Displacement/Rotation
*Boundary
_PickedSet4, 1, 1
_PickedSet4, 2, 2
** Name: top_fix Type: Displacement/Rotation
*Boundary
_PickedSet5, 1, 1
** ----------------------------------------------------------------
**
** STEP: Step-1
**
*Step, name=Step-1, nlgeom=NO, inc=1000
*Static
0.01, 1., 1e-05, 0.01
**
** BOUNDARY CONDITIONS
**
** Name: Top_U Type: Displacement/Rotation
*Boundary
_PickedSet6, 2, 2, 0.1
**
** OUTPUT REQUESTS
**
*Restart, write, frequency=0
**
** FIELD OUTPUT: F-Output-1
**
*Output, field
*Node Output
RF, U
**
** HISTORY OUTPUT: H-Output-1
**
*element output, elset=umatelem
SDV
*End Step
```

报错解决：

generate 似乎不能用于单个单元

Error in job fig_3-10-1: There are fewer than 2 items on a data line for which a generation sequence (start id, end id, optional increment number) is expected.

装配中应该有 output 的单元集合

Error in job fig_3-10-1: in keyword *ELEMENTOUTPUT, file "fig_3-10.inp", line 123: Unknown assembly set UMATELEM

Error in job fig_3-10-1: ELEMENT SET ASSEMBLYA_UMATELEM HAS NOT BEEN DEFINED

Job fig_3-10-1: Analysis Input File Processor aborted due to errors.

Error in job fig_3-10-1: Analysis Input File Processor exited with an error - Please see the fig_3-10-1.dat file for possible error messages if the file exists.

```
** ASSEMBLY
**
*Assembly, name=Assemblya
**
*Instance, name=plane_strain-1, part=plane_strain
*End Instance


**
*Nset, nset=_PickedSet4, internal, instance=plane_strain-1
 1, 2
*Nset, nset=_PickedSet5, internal, instance=plane_strain-1
 3, 4
*Nset, nset=_PickedSet6, internal, instance=plane_strain-1
 3, 4

*Elset, elset=umatelem, instance=plane_strain-1
3

*End Assembly
**
```

增加 umatelem

无语了 ， 教材上的应力是 x 方向的 他写 y 方向 ， 检查了半天。

另外弹性模量和能量释放率的单位考虑他也写错了。

目前代码在 Github 上实现了拉压能量分解，还没实现拉压应力分解，对于计算不卸载问题应该不会出错

Not enough data provided to generate the *ELSET: UMATELEM

Generate

Xxx, xxx,1

**这里不能有空行

不能有空行

关叉

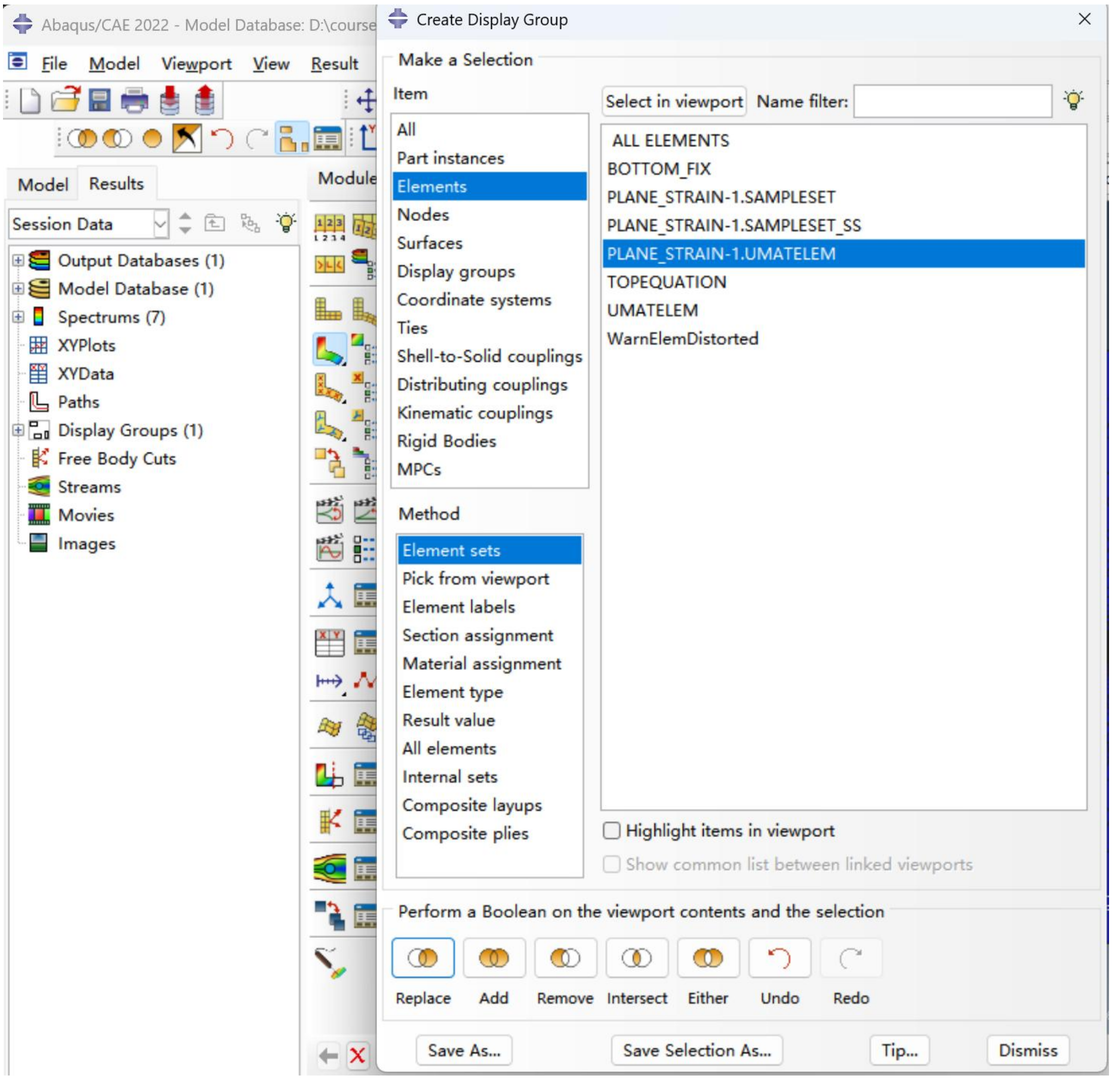

交错迭代需要很细的分析步，而拟牛顿法可以在粗分析步下得到较为准确的结果，但对模型要求很高，稍微改变一些参数就会无法收敛
